# Differential deficits in pattern- versus flash-visual evoked potentials in schizophrenia: relationship to subcortical visual systems, pulvinar nucleus and cognition

**DOI:** 10.3389/fnimg.2026.1833352

**Published:** 2026-06-18

**Authors:** Maria B. Aburto-Ponce, Kristin Micceri, Antigona Martinez, Daniel C. Javitt

**Affiliations:** 1 Nathan Kline Institute for Psychiatric Research, Orangeburg, NY, United States; 2 Department of Psychiatry, Columbia University Irving Medical Center, New York, NY, United States

**Keywords:** flash, pulvinar, schizophrenia, steady-state, transient, visual event-related potential

## Abstract

**Background:**

The human subcortical visual system is divided into distinct magnocellular, parvocellular and koniocellular pathways, which contribute differentially to specific aspects of early visual processing. Schizophrenia is associated with deficits in early-visual processing, especially involving N-methyl-D-aspartate receptor (NMDAR)-mediated non-linear gain within the subcortical magnocellular visual system. Nevertheless, methods for investigating the pathophysiological consequences remain limited. Flash-VEP can be obtained using either transient (tVEP) or steady-state (ssVEP) approaches. Flash stimuli also induce sustained reduction (“blocking”) of the posterior alpha rhythm. Red (vs. white) flash stimuli selectively suppress activity in magnocellular-recipient layers of primary visual cortex. Here, we investigated flash-VEP responses in schizophrenia, with emphasis on the potential utility for assessing selective pathophysiological involvement of the magnocellular and koniocellular pathways.

**Methods:**

We obtained flash-VEP from 28 healthy control and 27 schizophrenia participants to white and red stimuli across a range of stimulation rates, and pattern-VEP from 29 control and 22 schizophrenia participants. A subset (17 control/15 schizophrenia) participated in both studies. We also obtained fMRI to 6-Hz white and red stimuli in an additional sample of 14 control and 14 schizophrenia participants. fMRI analyses focused on both visual cortex and inferior pulvinar nucleus.

**Results:**

Schizophrenia participants showed increased flash-tVEP responses (*d* = 0.99, *p* < 0.001) despite significantly reduced pattern-tVEP (*d* = −0.99, *p* < 0.001). In addition, the ssVEP (photic driving) response was significantly reduced in schizophrenia, as reflected by reduced intertrial trial coherence (ITC) within the alpha frequency band (*d* = −0.78, *p* < 0.001). Alpha blocking was induced equivalently by white and red stimuli in HC, suggesting magnocellular involvement via the retinotectal system. The degree of blocking was significantly reduced in schizophrenia (*d* = −0.83, *p* = 0.003) and correlated significantly with neurocognitive impairment (r_p_ = 0.65, *p* < 0.001). fMRI studies showed reduced pulvinar activation (*d* = −1.1, *p* = 0.008), along with aberrantly increased dorsal cortical activation (*d* = 0.98, *p* = 0.019).

**Conclusion:**

The findings reinforce the importance of subcortical visual dysfunction as a driver of impaired neurocognition in schizophrenia and provide a scalable mechanism for assessment of early-visual dysfunction within the clinical setting. Deficit patterns are consistent with concepts of impaired magnocellular and koniocellular visual function affecting both thalamocortical and retinotectal system function in schizophrenia.

## Introduction

Deficits in early visual processing are a key component of schizophrenia and contribute to deficits in higher order cognition (Green et al., 2019; Javitt, 2023; Martinez et al., 2024; Ganse-Dumrath et al., 2025). The subcortical visual system in humans is divided into discrete magnocellular, parvocellular and koniocellular pathways, which project from the lateral geniculate nucleus of the thalamus (LGN) to primary visual cortex. These pathways are differentiated based on response patterns to stimulus properties such as contrast, spatial frequency and color (Martinez et al., 2024). Visual information also reaches cortex via the retinotectal system, which involves retinal projections to the superior colliculus and subsequently inferior subdivisions of the pulvinar nucleus of the thalamus (Grunert et al., 2021).

In schizophrenia, deficits are preferentially observed in response to stimuli that bias processing toward the magnocellular versus parvocellular visual systems, whereas the function of the koniocellular system has not been systematically investigated (Martinez et al., 2024). The visual processing deficits in schizophrenia are explained most parsimoniously by impaired *N*-methyl-D-aspartate receptor (NMDAR)-function within subcortical and early visual regions (Butler et al., 2005; Butler et al., 2007; Javitt, 2015). The physiological findings thus converge with recent reports of preferential and substantial reductions of NMDAR expression within primary and secondary visual cortical regions (Schoonover et al., 2024). Early visual processing deficits have been demonstrated most consistently in schizophrenia using methods such as visual backward masking (Green et al., 1994; Rassovsky et al., 2011; Schechter et al., 2003), contrast sensitivity (Butler et al., 2001; Butler et al., 2009) or pattern-induced visual evoked potentials (pattern-VEP) (Butler et al., 2007; Martinez et al., 2008, 2018). Such methods, however, require specialized equipment that cannot easily be implemented within the clinical setting.

In contrast, VEP to whole-field flash stimuli (flash-VEP) are routinely obtained using standard, clinical EEG equipment for assessment of epileptogenic potential among other purposes (e.g., Vranic-Peters et al., 2023). However, flash-VEP have been studied in schizophrenia to a relatively limited degree and have not incorporated contemporary neuro-oscillatory (“time-frequency”) approaches that decompose the responses according to underlying frequency bands and physiological processes thereby increasing the interpretability of the findings (Javitt et al., 2020). Key time-frequency measures include evoked power (“power of the average”), total power (“average of the power”), induced (non-time locked) power and intertrial coherence (ITC), also termed “phase locking.” Here, we investigated the potential utility of flash-VEP combined with single-trial time-frequency analysis, as potentially scalable biomarkers of early visual deficits in schizophrenia, as well as functional probes of underlying neurophysiological mechanisms.

Flash-VEP can be obtained using either transient (tVEP) or steady-state (ssVEP) approaches. Stimuli delivered at rates of ~1 Hz or lower (1 s ISI or longer) elicit a tVEP characterized primarily by increased power within the theta (4–7 Hz) and alpha (8–12 Hz) frequency bands. In non-human primates, generation of the tVEP (as opposed to the ssVEP) depends primarily upon parvocellular inputs to visual cortex mediated through non-NMDAR glutamate receptors (Schroeder et al., 1989; Schroeder et al., 1992; Kraut et al., 1985; Kraut et al., 1990; Schroeder et al., 1997). The parvocellular bias is further enhanced through the use of red stimuli, which induce enhanced activation within the ventral visual stream (Kraut et al., 1990; Chen et al., 2007). To the extent that the flash-tVEP has been studied in schizophrenia, it has mostly been found to be unimpaired (Connolly et al., 1983; Domino et al., 1979; Shagass et al., 1978), consistent with the limited magnocellular- and NMDAR involvement. This finding stands in contrast to the pattern-tVEP response in schizophrenia, which is reliably reduced especially by stimuli biased toward the magnocellular visual pathway (Butler et al., 2005; Schechter et al., 2005; Friedman et al., 2012).

For flash stimuli delivered at repetition rates of ~3/s or greater, the primary response consists of an ssVEP (also called “photic driving”) response that consists primarily of entrainment of ongoing alpha (8–12 Hz) posterior rhythms (Bayram et al., 2011). In the time domain, ssVEP appear as a continuous sinusoidal response entrained to the stimulation rate. ssVEP are thus traditionally analyzed only at the stimulation rate or harmonics thereof. In schizophrenia, when analyses are conducted in this way, deficits are observed primarily to stimuli presented at rates that fall within the theta or alpha-frequency bands (Butler et al., 2005; Butler et al., 2001; Rice et al., 1989; Jutai et al., 1984; Jin et al., 2000; Jin et al., 1995; Gruzelier et al., 1993; Murphy and Ongur, 2019; Kim et al., 2005; Schielke and Krekelberg, 2022; Brenner et al., 2009). In monkeys, the photic driving response elicited by repetitive visual stimuli is significantly reduced by sub-anesthetic doses of phencyclidine (Domino, 1964), suggesting underlying NMDAR involvement.

Finally, flash stimuli are known to cause sustained reduction (“blocking”) of the ongoing posterior alpha rhythm. This effect was first reported almost 100 years ago (Skelley et al., 2009), shortly after the initial description of the EEG itself (Berger, 1929). Alpha blocking deficits were first reported in schizophrenia shortly thereafter (MacMahon and Walter, 1938) and were then replicated repeatedly in the classic literature (Liberson, 1945; Blum, 1957; Salamon and Post, 1965). However, the alpha blocking deficits have not been systematically studied using modern data-analytic approaches. Similar to the photic driving response, alpha activity over visual cortex is reliably reduced by psychotomimetic doses of NMDAR antagonists (Vlisides et al., 2018; McMillan et al., 2020). In addition, recent computational models argue that alpha blocking is best explained by NMDAR-mediated prolongation of alpha oscillatory dynamics (Liley and Muthukumaraswamy, 2020), such that in the presence of an NMDAR antagonist, sensory stimulation will have a reduced effect on ongoing alpha activity.

In addition to VEP, we conducted a visual fMRI study. In fMRI, we have previously observed deficits in cortical activation to transiently presented low-, but not high-, spatial frequency Gabor patches (Martinez et al., 2012), as well as functional reorganization, such that dorsal stream visual regions in schizophrenia show unexpected hypersensitivity to parvocellular-biased (high-spatial frequency) inputs (Martinez et al., 2008). We have also observed fMRI activation deficits in pulvinar nucleus to motion stimuli (Martinez et al., 2019, 2021) as well as correlations between impaired pulvinar activation and impaired 10-Hz ssVEP responses (Martinez et al., 2018). In contrast, we have not observed between-group differences in cortical responses during ssVEP stimulation (Calderone et al., 2013). Here, we evaluated activation patterns in both the dorsal/ventral visual cortical regions and in the subcorticual retinotectal system including pulvinar nucleus.

Our goals in this study were two-fold: first, to evaluate the sensitivity of neuro-oscillatory changes induced by repetitive flash stimuli as indices of early visual processing dysfunction in schizophrenia and second, using multimodal imaging, to further evaluate the integrity of specific subcortical visual pathways relative to NMDAR theories in schizophrenia. We hypothesized that schizophrenia would be associated with deficits in both ssVEP (photic driving) responses and alpha blocking, but with intact tVEP response; and that ssVEP and alpha-blocking deficits would correlate with impairments in higher order neurocognition.

## Methods

### Participants

Fifty-five individuals participated in the flash-VEP portion of the study, including 27 individuals with schizophrenia and 28 healthy controls as diagnosed using the Structured Clinical Interview for DSM-5. Groups were similar in mean age (controls: 36.5 ± 10.6, schizophrenia:39.1 ± 10.3 yr), and gender distribution (control:7F,20M; schizophrenia:1F,25M). For the schizophrenia group, positive and Negative Syndrome Scale (PANSS) (Kay et al., 1986) mean scores were 74.1 ± 13.4 (total), 20.1 ± 5.3 (positive) and 18.4 ± 5.3 (negative). Mean length of illness was 15.4 ± 9.3 yrs. Mean antipsychotic dose (chlorpromazine equivalents) was 921.7 ± 785.7. Cognitive function was assessed using the MATRICS consensus cognitive battery (MCCB) (Nuechterlein et al., 2008).

Data were also analyzed from a parallel study investigating mechanisms of feature attention dysfunction in schizophrenia. Total participants included 22 schizophrenia and 29 control individuals, of whom 17 control and 15 schizophrenia individuals also participated in the flash-VEP study. Mean ages for the HC and Sz groups were 36.3 ± 12.90 and 37.4 ± 10.3, yr., respectively. Gender distribution was 10F/19M for controls vs. 1F/21M for the schizophrenia group. Mean PANSS scores were 11.9 ± 4.9 (positive) and 15.2 ± 7.0 (negative). For the schizophrenia group, mean antipsychotic dose was 756.1 ± 457.9 CPZ equivalents and mean length of illness was 13.4 ± 9.5 yrs.

A final sample of twenty-eight individuals (14 schizophrenia, 14 control) participated in a follow-up fMRI component. These individuals showed similar mean age (controls: 38.4 ± 10.2; schizophrenia: 33.2 ± 9.3 yr) and gender (control: 2F, 12M; schizophrenia: 2F, 12M). For the schizophrenia group, mean illness duration was 11.4 ± 8.6 yrs. and mean antipsychotic dose was 541.6 ± 392.4 CPZ equivalents.

For all studies, participants were recruited from inpatient units and residential care facilities associated with Nathan Kline Institute for Psychiatric Research (NKI). The study was approved by the NKI institutional review board. All participants signed consent forms for their participation in this study following full explanation of study procedures.

### Stimulus presentation

Flash-VEP were obtained in response to white and red photic flash stimuli presented in bursts of 10 s at rates of 1, 3, 6, 9, and 12 Hz, with pauses of 5 s between bursts, using the LED-based flash device EELITE (CognitraceMT, Enschede, Netherlands), and delivered by the software EEVOKED. The LED device was placed 100 cm from the subject. For the red stimuli, a red filter (Roscolux #19, Rosco Laboratories Inc.) (Chen et al., 2007) covered the LED. For the white stimulus, a neutral density filter (one log unit) was used to provide equivalent luminance. These filters permit 20% (red only) and 10% (cross-color) transmission, respectively.

For pattern-tVEP, stimuli consisted of grey and red checkerboard stimuli with center cutouts to minimize eye movements presented with 100-ms duration. Stimuli were presented in the context of a feature-attention study in which stimuli were presented in both attended and unattended contexts, with mean stimulus onset asynchrony (SOA) of 1,000 ms. Here, we analyzed responses to unattended stimuli only.

During fMRI, stimuli were delivered using an LED goggle system, presented at central fixation with a mean stimulation rate of 6 Hz. White and red flickering stimuli were presented relative to a uniform gray background in separate runs.

### ERP recording and pre-processing

In order to calculate ERP, the ongoing electroencephalogram (EEG) was recorded on an FDA 510 K-cleared EEG system (Advanced Neuro Technology, ANT, Enschede, Netherlands) using a custom Waveguard cap containing 64 equally spaced electrodes covering the whole head from slightly above the eyebrows to below the inion (Woldorff et al., 2002). All channels were referenced to a frontal midline (E1Z) channel. Impedances of all electrodes were kept below 5 kΩ throughout the experiment. Eye movements were monitored with bipolar electrodes placed on the left and right outer canthi to record the horizontal EOG. Data were acquired at a sampling rate of 512 Hz and filtered offline using half-amplitude cutoffs of 0.1 and 46 Hz. ERP were analyzed in the time-frequency (“spectral”) domain using a three-cycle Morlet wavelet decomposition (Javitt et al., 2020) computed over 73 frequencies ranging from 0.73 to 53.5 Hz, incremented logarithmically (Lakatos et al., 2005).

### ERP analyses

For ERP, analyses focused primarily on responses within the theta (4–7 Hz) and alpha (8–12 Hz) frequency bands. Alpha-frequencies were divided into lower (6–10 Hz) and higher (10–12 Hz) components. For tVEP, the latency range of interest was determined based on combined schizophrenia and control data to avoid circularity (Kriegeskorte et al., 2009). For photic driving, modulations in alpha ITC were computed by extracting the alpha frequency band, computing a Fast Fourier Transform (FFT) and calculating peak values in a window of ±0.5 Hz around the stimulation rates (3,6,9,12-Hz). For alpha blocking, statistical analyses were performed on the change in power during the initial 5-s following stimulus onset related to the 5-s pre-stimulus baseline. Baseline activity was collapsed across the white- and red- stimulation blocks. Between-group differences were determined using univariate or repeated-measures ANOVA as appropriate.

### fMRI acquisition and pre-processing

fMRI data were collected on a Siemens 3 T TIM-Trio scanner (Siemens Corp., Erlangen, Germany). A high-resolution structural image was acquired per participant using an MP-RAGE sequence (spatial resolution = 1 mm isotropic, repetition time (TR) = 2000 ms; echo time (TE) = 3.5 ms; flip angle 8°, 192 slices). Echo-planar images (EPIs) (2.5 mm isotropic, TR = 2000 ms, TE = 30 ms, flip angle 80°) were acquired on 36 contiguous slices in the axial plane. Functional data were pre-processed using a combination of Analysis of Functional NeuroImages (AFNI) (McMillan et al., 2020) and Surface Mapping (SUMA) software (Saad and Reynolds, 2012). Preprocessing consisted of removal of despiking (3dDespike), temporal alignment, identification of motion outliers and scaling of blood-oxygen-level-dependent (BOLD) values to mean percent signal change (Reynolds et al., 2024). Cortical data was spatially smoothed with a 6 mm full-width-at-half-maximum Gaussian kernel. For each participant, the cortical surface was rendered using FreeSurfer (Fischl, 2012). Analyses were carried out on gray-matter ordinates of each individual cortical surface aligned to the 141-fsaverage standard mesh. Cortical data was sampled to the Human Connectome Project (HCP-MMP) multimodal cortical parcellation atlas (Glasser et al., 2016) resampled to fsaverage. For subcortical structures, parallel analyses were carried out in volumetric space registered to the MNI-152 template brain.

A general linear model implemented in AFNI’s *3dDeconvolve* was used to analyze the contrast of red versus white stimuli in each participant. Regions of interest (ROIs) were defined based on HCP-MMP parcellation of the dorsal and ventral visual streams with the dorsal ROI comprising six parcels (V3A, V3B, V6, V6A, V7, IPS) and the ventral ROI comprising seven parcels (V8, VVC, PIT, FFC, VMV1, VMV2, VMV3). Mean beta values were extracted from each ROI, as well as from the pulvinar, which was defined using an anatomically segmented mask based on the MNI template. Group-wise analyses were carried out on mean beta values from these 3 regions. Significance levels were set to a (corrected) *p* = 0.01.

### Statistical analyses

Data were analyzed by univariate, multivariate or mixed model regression as appropriate, with diagnostic group as a between-subject factor and stimulus type as a within-subject factor. For correlational analyses conducted across groups, partial correlations were conducted controlling for group. Additional correlations were conducted in the schizophrenia group alone. For all analyses, a pre-set alpha level of *p* < 0.05 was used for significance. Effect sizes are reported as Cohen’s d, calculated as 2*(η^2^/(1 − η^2^)). By convention, cutoffs for small, moderate and large effects are 0.2, 0.5 and 0.8 (Cohen, 1988).

## Results

### tVEP

**Figure 1:**
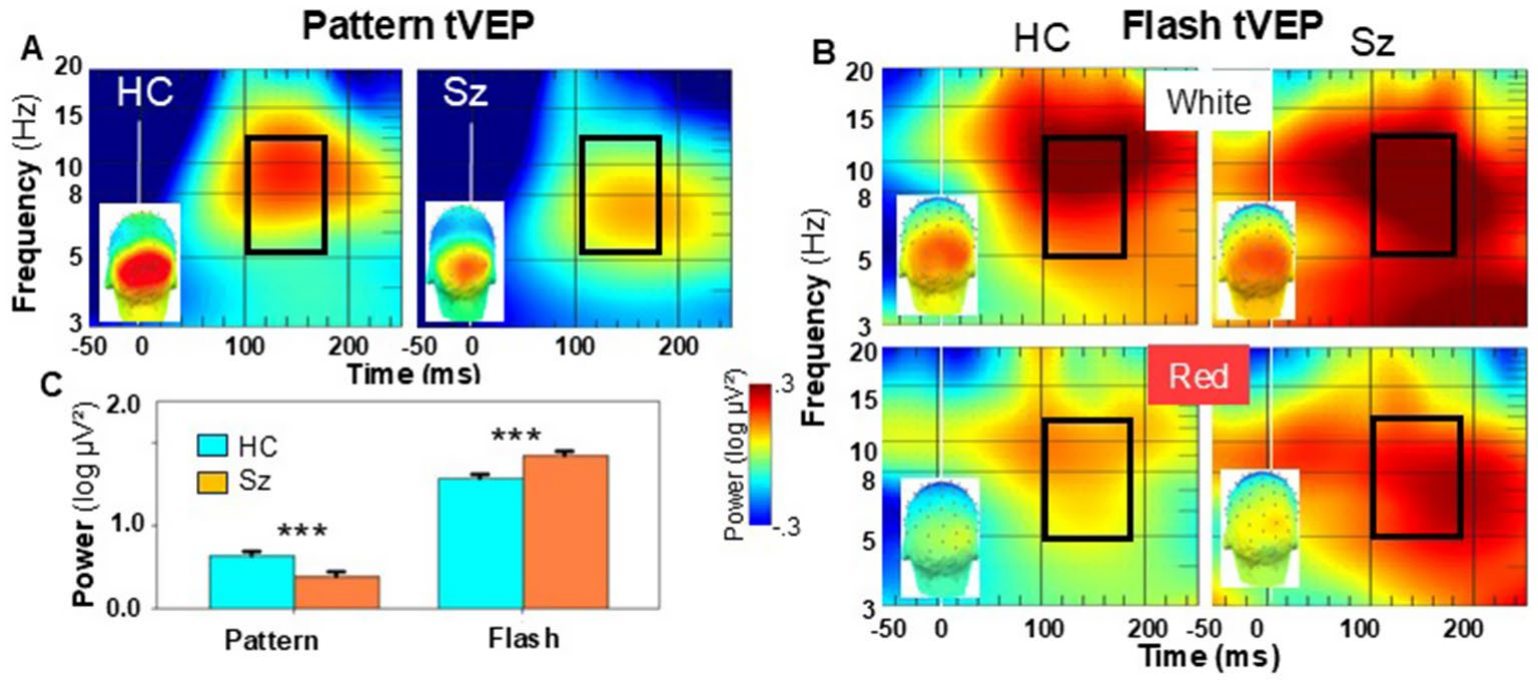
Comparative responses to **(A)** pattern and **(B)** flash-tVEP between schizophrenia (SZ) and healthy control (HC) participants. **(C)** Mean responses for pattern- and flash-tVEP by group. ***p < 0.001 HC vs. Sz.

Both the pattern- (Figure 1A) and flash-tVEP (Figure 1B) were manifest primarily as increases in power across the theta and alpha frequency ranges in the 100–175 ms latency range. As expected, pattern tVEP amplitudes were significantly reduced in schizophrenia across colors (F_1,49_ = 11.6, *p* = 0.001, *d* = 0.97), with no significant group X color interaction (F_1,49_ = 1.26, *p* = 0.3) (Table 1). In contrast, flash-tVEP response were significantly increased across colors (F_1,53_ = 12.6, *p* < 0.001, *d* = 0.98), also with no significant group X color interaction (F_1,53_ = 0.45, *p* = 0.5).

**Table 1:** Response magnitudes of the transient (tVEP) and steady-state (ssVEP) visual evoked potential responses, and stimulus-induced *α*-blocking.

Stimulus	Rate	Control		Schizophrenia		*t*	*p*	*d*
Color		Mean	SD	Mean	SD			
*Pattern tVEP (log uV^2^)* ^a^								
Gray	1 Hz	0.62	0.26	0.36	0.29	**3.40**	**0.001**	**0.96**
Red	1 Hz	0.64	0.25	0.43	0.24	**3.14**	**0.003**	**0.89**
*Flash-tVEP (log uV^2^)* ^b^								
White	1 Hz	1.58	0.33	1.87	0.25	**3.67**	**<0.001**	**0.99**
Red	1 Hz	1.56	0.34	1.82	0.26	**3.12**	**0.002**	**0.86**
*ssVEP (ITC, x10^−3^)* ^b^								
White	1 Hz	0.27	0.41	0.13	0.22	−1.57	0.12	−0.42
	3 Hz	**3.40**	**4.18**	**1.46**	**1.67**	**−2.25**	**0.028**	**−0.63**
	6 Hz	**5.03**	**5.82**	**1.74**	**1.81**	**−2.81**	**0.007**	**−0.76**
	9 Hz	0.43	0.64	0.35	0.54	−0.47	0.64	−0.13
	12 Hz	0.17	0.25	0.60	1.46	1.52	0.13	0.40
Red	1 Hz	0.11	0.17	0.12	0.23	0.24	0.81	0.07
	3 Hz	1.35	2.44	1.50	2.65	0.21	0.83	−0.06
	6 Hz	1.89	2.45	1.34	1.80	−0.94	0.35	0.25
	9 Hz	0.46	1.05	0.46	0.61	−0.02	0.98	0.01
	12 Hz	0.18	0.32	0.21	0.40	0.28	0.78	−0.08
*α blocking (log uV^2^)* ^b^								
Comb-Baseline	Comb	2.80	0.36	2.94	0.29	−1.61	0.11	0.44
Comb-During	Comb	**2.62**	**0.31**	**2.86**	**0.27**	**−3.09**	**0.003**	**0.83**

a Sample size: 29 HC, 22 Sz.

b Sample size: 28 HC, 27 Sz.

**Figure 2:**
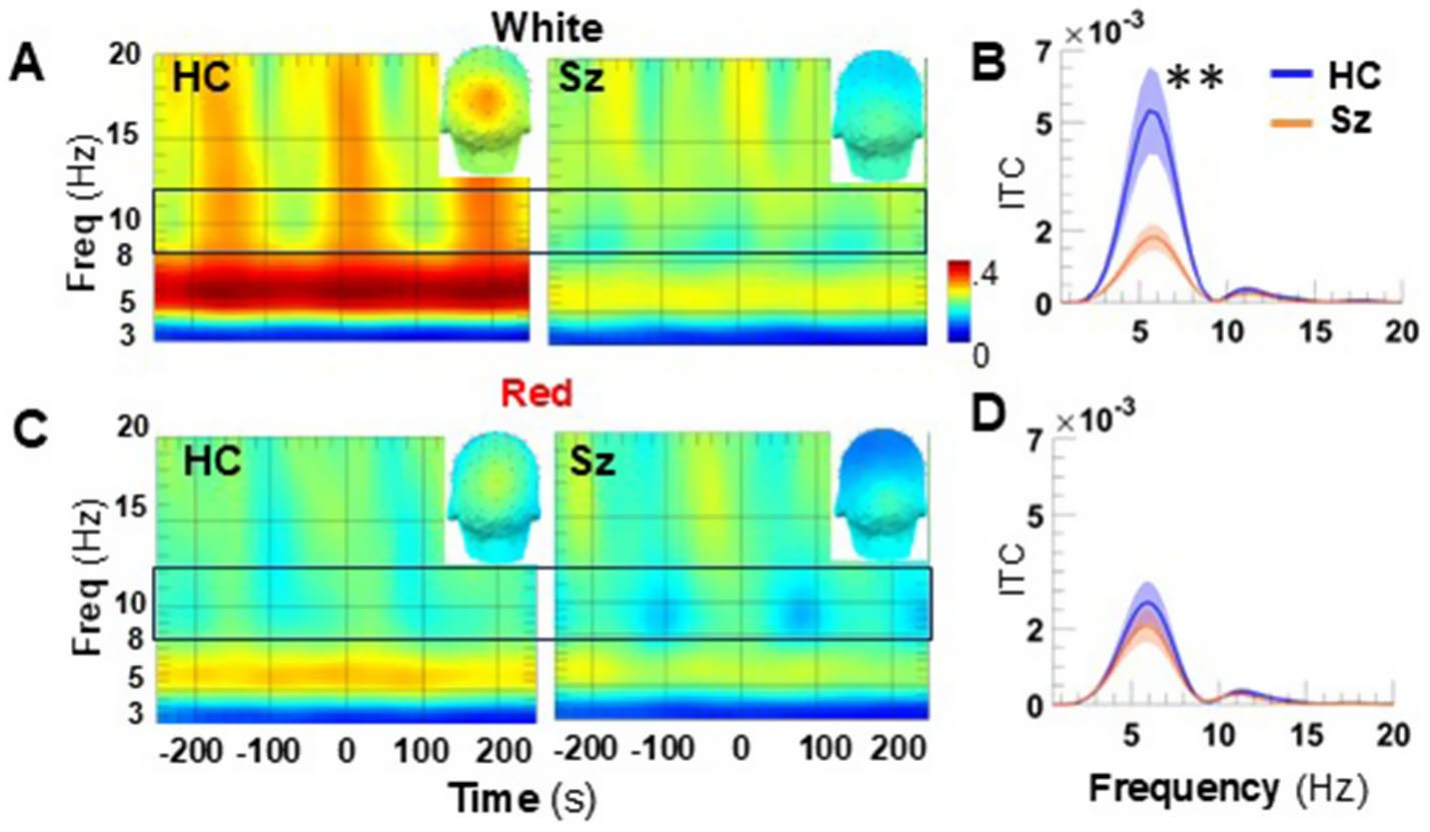
Photic driving response to white and red stimuli in healthy control (HC) and schizophrenia (Sz) participants. **(A)** Time-frequency plots of intertrial coherence (ITC) to 6 Hz white flash stimuli across groups. Boxed area shows the alpha (8–12 Hz) frequency range. **(B)** Mean fast-Fourier transform (FFT) of alpha ITC values (±SEM) to white flash stimuli, showing differential modulation at the stimulation rate in HC vs. Sz individuals, **p < 0.01. **(C)** Time-frequency plots to red flash stimuli. **(D)** FFT of ITC values (±SEM) to red flash stimuli.

**Figure 3:**
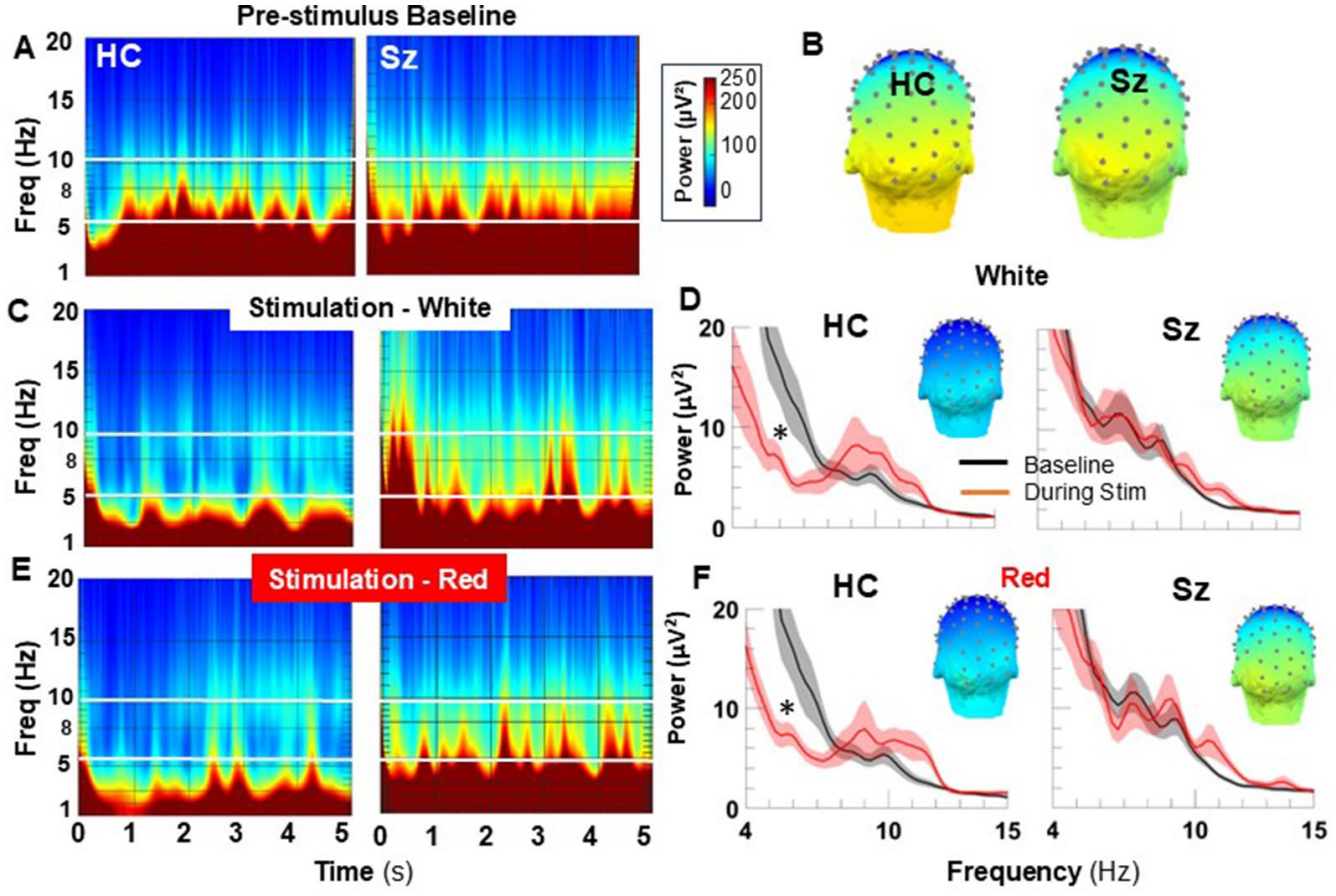
Time-frequency (TF) plots of ongoing power at baseline versus during stimulation for healthy control (HC) and schizophrenia (Sz) participants. **(A)** TF plots of pre-stimulation activity. Box represents the 5–10 Hz frequency range. **(B)** Head maps showing scalp distribution of activity. **(C)** TF maps of activity during white flash stimulation (collapsed across stimulation rates) showing reduced suppression in Sz. **(D)** Fast-Fourier transform (FFT) of spectral power at indicated spectral frequencies during white stimulation versus baseline for HC. Lines are mean ±standard error of the mean. *p < 0.05 baseline vs. during stimulation. **(E)** TF plots during red flash stimulation. **(F)** FFT of spectral power at indicated spectral frequencies.

tVEP are assessed as power *(log uV^2^)* as shown in Figure 1. ssVEP are assessed as intertrial coherence (ITC) as shown in Figure 2. α-blocking is assessed as ongoing power *(log uV^2^)* as shown in Figure 3.

Values in bold mean significant differences between groups (*p* < 0.05).

A mixed model regression across both stimulus types (pattern, flash) collapsed across color showed a highly significant group X stimulus-type interaction (F_1,102_ = 23.9, *p* < 0.001, *d* = 0.97) (Figure 1C) consistent with differential magnocellular vs. parvocellular dysfunction. Finally, both the decrease in the pattern-tVEP (F_1,30_ = 7.01, *p* = 0.013, *d* = 0.97) and the increase in the flash-tVEP (F_1,29_ = 12.7, *p* = 0.001, *d* = 1.3) response were significant, as was the stimulus type X group interaction (F_1,29_ = 24.9, *p* < 0.001, d = 1.9).

### Photic driving

The amplitude of the photic driving response was quantified by calculating the modulation of the alpha-ITC at the stimulation rate for each stimulation rate (Table 1). For white stimuli, across stimulation-rates, there was a highly significant main effect of group (F_1,53_ = 7.67, *p* = 0.008, *d* = 0.78) along with a significant main effect of stimulation rate (F_3,51_ = 10.63, *p* < 0.001, *d* = 0.91) and stimulation-rate X group interaction (F_3,51_ = 3.83, *p* = 0.015, *d* = 0.54). Across rates, the greatest reduction in was observed to 6-Hz stimulation (t = 2.81, df = 53, *p* = 0.007, *d* = 0.76) (Figures 2A,B).

As expected, for red stimuli, there was no significant between-group difference (F_1,53_ = 0.14, *p* = 0.71) or group X stimulation-rate interaction (F_3,51_ = 0.37, *p* = 0.78) (Figures 2C,D). When white and red responses were entered into a simultaneous ANOVA, the main effect of color (F_1,53_ = 11.3, *p* = 0.001, *d* = 0.92) and the color X group (F_1,53_ = 6.86, *p* = 0.011, *d* = 0.72) interactions were statistically reliable. As opposed to the significant difference in ITC, there were no significant differences in alpha power across colors or stimulation rate (F_1,53_ = 1.46, *p* = 0.2).

### Alpha blocking

Alpha-blocking was calculated for the first 5 s following stimulus onset relative to the 5 s no stimulation baseline collapsed across stimulation rates (Figure 3). Pre-stimulation amplitudes were not significantly different between groups over the theta and alpha frequency ranges. Nevertheless, activity in the 5–10 Hz range was significantly larger across white and red stimulation (F_1,53_ = 9.53, *p* = 0.003, *d* = 0.84) as was the pre-post difference covaried for baseline (F_1,52_ = 7.69, *p* = 0.008, *d* = 0.77) (Table 1).

### Correlations

**Figure 4:**
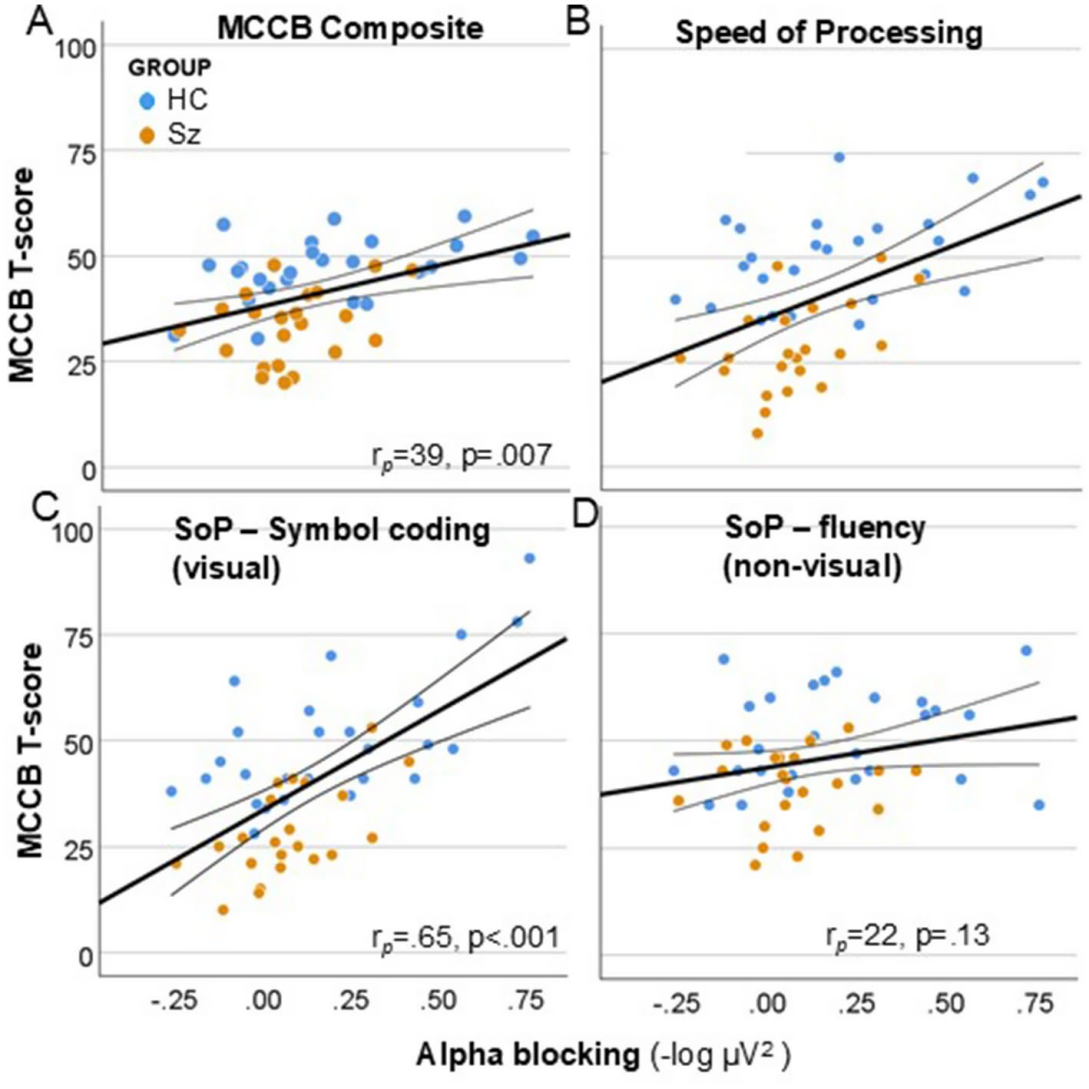
Correlation plots for alpha blocking versus indicated neurocognitive measures across healthy control (HC) and schizophrenia (Sz) groups. **(A)** Alpha blocking vs. MCCB composite score. **(B)** Alpha blocking vs. speed of processing (SoP) domain score. **(C)** Alpha blocking vs. symbol coding test (visual SoP). **(D)** Alpha blocking vs. category fluency test (non-visual SoP). Partial correlation values (r_p_) reflect correlations between variables covaried for group status.

Correlational analyses were conducted both across groups, covaried for group status, as well as within the schizophrenia group alone. MCCB composite T-scores (*p* < 0.001) were significantly reduced in schizophrenia (23.2 ± 12.0) vs. controls (44.1 ± 11.2). Across groups, deficits in alpha blocking correlated significantly with the MCCB composite score (r*_p_* = 0.39, *p* = 0.007) with no significant difference in the slope of the relationship between groups (F_1,44_ = 0.23, *p* = 0.6) (Figure 4A).

Significant independent correlations were observed for Speed of Processing (r*_p_* = 0.49, *p* < 0.001) (Figure 4B), Visual Learning (r*_p_* = 0.47, *p* < 0.001), Verbal Learning (r*_p_* = 0.54, *p* < 0.001) and Working Memory (r*_p_* = 0.34, *p* = 0.021). Both Speed of Processing and Working Memory contain measures that have primarily visual and primarily non-visual components.

For Speed of Processing, a significant correlation was observed for the visual component (Symbol-Coding, r*_p_* = 0.65, *p* < 0.001) but not for the non-visual component (Category Fluency, r*_p_* = 0.18, *p* = 0.22) (Figures 4C,D). Similarly, for Working Memory, a significant correlation was observed for Spatial Span (r*_p_* = 0.34, *p* = 0.02) but not Letter-Number Span (r*_p_* = 0.22, *p* = 0.13), suggesting a preferential relationship to visually based neurocognitive functions.

### fMRI

**Figure 5:**
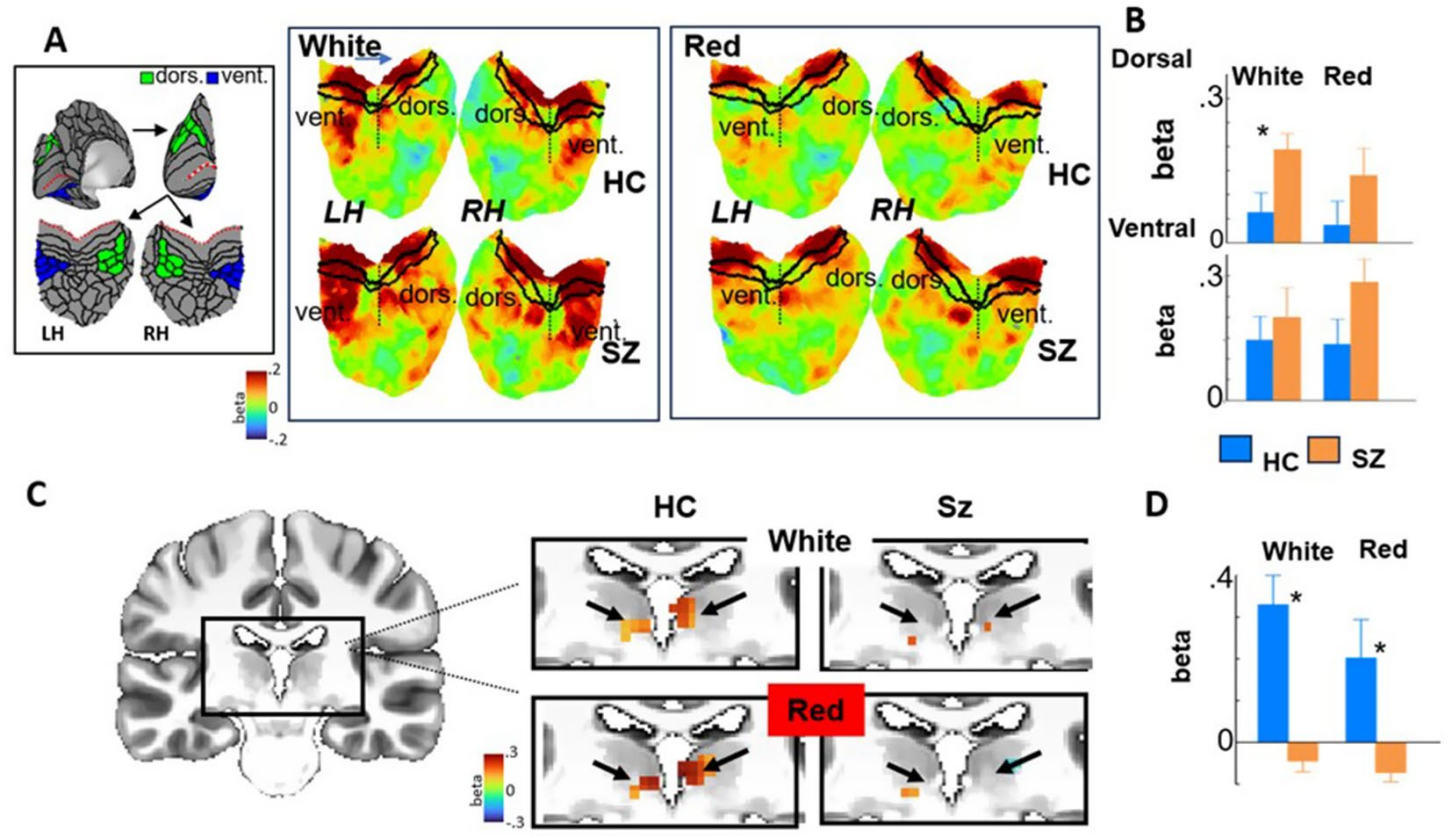
fMRI activation patterns during repetitive white versus red stimulation across healthy control (HC) and schizophrenia (Sz) participants **(A)** Schematic of flattening procedure is shown on left. Original cortical surface → unfolded occipital surface → flattened occipital surface via calcarine cut (red-dotted line). Dorsal (green) and ventral (blue) parcels from the HCP-MMP atlas are indicated. Cortical activation patterns to white and red stimuli are shown on flattened left (LH) and right (RH) hemisphere surfaces. **(B)** Bar plots showing cortical activations (top: dorsal, bottom: ventral) across groups (HC vs SZ), *p < 0.05, **p < 0.01; **(C)** Subcortical activation patterns across groups. **(D)** bar plots showing reduced pulvinar nucleus activation in Sz. *p < 0.05 HC vs. Sz.

In order to evaluate potential underlying substrates for the ERP effects, a second cohort of patients was studied using fMRI and 12-Hz white and red stimulation. As predicted, controls showed significantly greater activation in ventral vs. dorsal cortical regions (F_1,13_ = 7.60, *p* = 0.016, *d* = 1.53), whereas this difference was absent in schizophrenia (F_1,13_ = 1.49, *p* = 0.24), reflecting a significantly larger response in schizophrenia to white stimuli in the dorsal visual stream (F_1,26_ = 6.21, *p* = 0.02, *d* = 0.98) (Figures 5A,B).

Within pulvinar nucleus, controls participants showed significant activation to both white [*t* (13)=2.45, *p* = 0.029, *d* = 0.66] and red [*t* (13)=2.19, *p* = 0.048, *d* = 0.58] stimuli, whereas schizophrenia participants did not (both *p* > 0.20), leading to a highly significant main effect of group (F_1,26_ = 8.14, *p* = 0.008, *d* = 1.12) (Figures 5C,D). When analyzed across regions (dorsal, ventral, pulvinar) and colors, the group X region effect was significant (F_2,25_ = 7.04 *p* = 0.003, *d* = 1.5) reflecting the enhanced cortical vs. reduced subcortical (pulvinar) activation in schizophrenia.

### Control analyses

No significant correlations were observed between age, sex, medication dose of length of illness for either the ERP or fMRI measures, following correction for multiple comparisons. As expected based upon the number of correlations, one correlation was nominally significant (white, 6 Hz ssVEP versus sex, r = 0.33, *p* < 0.05). Nevertheless, the between-group difference remained significant following covariation for sex (F_1,52_ = 4.77, *p* = 0.034).

## Discussion

Deficits in early visual processing in schizophrenia were first demonstrated in the early 1900’s (Busch, 1908; Donde et al., 2019), have been replicated extensively since that time (Martinez et al., 2024) and shown to contribute to overall cognitive dysfunction (Martinez et al., 2024; Ganse-Dumrath et al., 2025; Rassovsky et al., 2011). Moreover, recent post-mortem studies demonstrate a caudal-rostral gradient of abnormalities in the brain such that histological disturbances in visual cortex are significantly more extensive than those found in more anterior brain regions cortex. Abnormalities include reduced volume and neuron number (Dorph-Petersen et al., 2007), reduced GABA biomarker expression (Fish et al., 2021), reduced energy expenditure (Kimoto et al., 2022) and reduced NMDAR expression (Schoonover et al., 2024). Significant volume reduction is also observed in the thalamic pulvinar nucleus (Byne et al., 2009; Aleman-Gomez et al., 2020; Huang et al., 2024). Nevertheless, methods for assessment of early visual processing dysfunction in schizophrenia remain limited.

In this study, we used a flash-VEP paradigm combined with single-trial, time-frequency data analysis, as a complement to traditional pattern-VEP approaches. Despite the simplicity of the stimulation procedure, we confirm earlier findings of robust deficits in early visual processing in schizophrenia that preferentially involve the magnocellular-, and potentially koniocellular-, visual pathways and that correlate extensively with the degree of overall neurocognitive impairments (Martinez et al., 2024). Using fMRI, we also provide further evidence of pulvinar nucleus dysfunction in schizophrenia related to impaired visual processing and social cognition (Martinez et al., 2018, 2021). Based on these findings, we propose that both the photic driving (ssVEP) and alpha-blocking responses to repetitive flash stimulation (1) represent robust and computationally tractable signatures of early visual processing dysfunction in schizophrenia, (2) correlate with overall levels of cognitive impairment, and (3) further support concepts of subcortical contributions to cortical-level dysfunction in schizophrenia.

### Visual system physiology

The early visual system originates in the retina and projects to cortex via the retinogeniculate and the retinotectal systems. The retinogeniculate system is divided into discrete magnocellular, parvocellular and koniocellular divisions, which target discrete layers of the lateral geniculate nucleus (Martinez et al., 2024; Solomon, 2021). Both the magnocellular and parvocellular pathways receive input from long (red)-and medium (green)- wavelength cones in retina but with additivity versus opponency. Thus, magnocellular neurons respond most strongly to achromatic stimuli. In contrast, parvocellular neurons are uniquely sensitive to color contrast. Both the magnocellular and parvocellular pathways primarily target layer 4 of cortex and serve as driver inputs (Sherman and Guillery, 2002; Sherman, 2017).

In contrast, koniocellular cells are preferentially activated by short wavelength (blue/yellow) opponency cones and primarily innervate superficial layers of V1, where they act as modulator inputs (Hendry and Reid, 2000). In addition, approximately 10% of magnocellular and koniocellular ganglion cells target non-geniculate subcortical relays including the superior colliculus and the inferior portion of the pulvinar nucleus via the retinotectal system (Grunert et al., 2021). The inferior pulvinar nucleus, in turn, primarily targets superficial layers within cortex and thus serves as an extended matrix system to modulate ongoing oscillatory activity within the early visual system (Huo et al., 2019; Kwan et al., 2019).

### Flash-tVEP

The flash-tVEP in cortex has been extensively studied in non-human primates and has been shown to depend primarily on parvocellular input to cortex. The selectivity is due to local inhibitory feedback within the superficial layers of primary visual cortex that selectively suppress magnocelluar input (Kraut et al., 1990). Thus, when the local inhibitory processes are inhibited through local administration of the GABA_A_ receptor inhibitor bicuculline, a large, localized *increased* response is observed to flash activation within magnocellular-recipient layers of cortex. This increase, in turn, is inhibited by simultaneous administration of the NMDAR antagonist ketamine. In contrast, ketamine has little effect on the flash-tVEP response in the absence of GABAergic inhibition (Schroeder et al., 1992). The parvocellular bias can be further increased through the use of red stimuli, which preferentially activate the ventral visual stream (Chen et al., 2007).

In our study, as predicted, schizophrenia was associated with an enhanced flash-tVEP response, despite impaired pattern-tVEP responses within the same study population (Figure 1). Schizophrenia was also associated with enhanced fMRI responses in visual sensory regions to flash stimuli, especially red (Figure 5A). In contrast, we have previously shown reductions in primary cortex and dorsal stream visual regions to pattern stimuli (Martinez et al., 2018, 2019). Similarly, we have previously observed that schizophrenia is associated with a remapping of visual regions, such that dorsal stream cortex receives aberrant high-spatial frequency input via the parvocellular system that replaces the normal low-spatial frequency magnocellular driven input (Martinez et al., 2008). Here, we suggest that the flash-tVEP, especially to red stimuli, may serve as a sensitive index of the pathological remapping of cortex, including increased parvocellular input into dorsal stream pathways.

### Photic driving (ssVEP) response

The ssVEP, which is elicited by rapidly presented stimuli, is distinguished from the tVEP in that it consists purely of phase-reset of ongoing oscillatory rhythms, with no alteration in induced power or associated cortical activation (Bayram et al., 2011). The mechanisms underlying this phase reset are incompletely understood even within the normative population. The photic-driving response is typically calculated by applying an FFT to ongoing EEG activity and then analyzing power either at the stimulation rate or harmonics thereof. When analyzed using this approach, deficits in schizophrenia are significant but often statistically small (Schielke and Krekelberg, 2022). Here, we refine this approach by first extracting the ITC (phase-reset) component within the alpha-frequency band using single-trial decomposition (Figure 2A), converting the extracted ITC response into a continuous variable (“pseudo-ERP”) (Supplementary Figure 1) and then performing the FFT on the extracted alpha-band response (Figure 2B). Using this approach, we show a large between-group difference to repetitive white-flash stimuli both across stimulation rates (*d* = 0.78) and at the 6-Hz stimulation rate specifically.

Our study was not designed to specifically evaluate koniocellular visual pathway function. Nevertheless, the differential response to white versus red stimuli and the lack of a power change accompanying the ITC response are consistent with koniocellular involvement given the insensitivity of the koniocellular system to long-wavelength (red) stimuli and its innervation only of superficial layers of cortex. To the extent that the koniocellular system does mediate the photic driving response, this study would be the first neurophysiological demonstration of koniocellular impairment in schizophrenia, although one prior study has shown reduced contrast-sensitivity to konio-biased stimuli (Timucin et al., 2017). At present, little is known about the role of NMDAR in koniocellular system function. Nevertheless, ketamine has been shown to suppress the ssVEP response in non-human primates (Schielke and Krekelberg, 2022; Domino, 1964), suggesting NMDAR involvement in koniocellular, as well as magnocellular, pathway function. Future studies using short-wavelength (blue) flashes are required to further evaluate the mechanisms of driving ssVEP responses across both normative and schizophrenia populations, and in characterizing ssVEP impairments in schizophrenia.

### Alpha blocking and pulvinar dysfunction

Finally, the present study represents the first quantitative study of alpha blocking in schizophrenia. Alpha blocking refers to the ability of flash stimuli to suppress ongoing posterior alpha activity independent of responses to each individual stimulus. Deficits in alpha blocking were first documented in schizophrenia in the pre-antipsychotic era (MacMahon and Walter, 1938; Liberson, 1945; Blum, 1957; Salamon and Post, 1965) using qualitative approaches in which researchers manually counted the number of alpha waves during the pre- vs. post-stimulation period. Here, we replicate and extend these findings using more modern analytic approaches.

The ongoing alpha rhythm is usually characterized in the 8–12 Hz frequency range. However, recent cluster-analytic studies suggest that it may be subdivided into two discrete components: a higher- (10–12 Hz) frequency component that is most prominent over the dorsal visual stream, and a lower- (6–10 Hz) frequency component that is most prominent over the ventral visual stream (Barzegaran et al., 2017). In controls, flash-induced alpha blocking was most prominent within the 6–10 Hz range over ventral cortex, and was virtually absent in schizophrenia (Figures 3D,F).

The deficits in schizophrenia moreover correlated with deficits across several neurocognitive domains including overall cognition and speed of processing (Figure 4), emphasizing the role of dynamic alpha modulation as a key neurophysiological process related to cognitive processing. Moreover, within the speed of processing domain, the correlation was significant with the symbol coding test, which relies extensively on visual processing; but not the category fluency test, which has no visual component. Thus, stimulus-induced alpha-blocking in the EEG may specifically index the failure to engage visual systems during visual tasks in schizophrenia. Our present findings regarding flash-induced suppression also mirror previous findings from our group (Martinez et al., 2015, 2019) and others (e.g., Ramsay et al., 2024) showing impaired alpha-modulation during cognitive task performance related to cognitive dysfunction in schizophrenia.

Although the mechanisms underlying alpha blocking are not fully understood, the pulvinar nucleus is considered a key structure mediating alpha-frequency interactions between thalamus and cortex and may mediate both “bottom up” and “top down” modulations of ongoing alpha activity (Cortes et al., 2020; Kastner et al., 2020). We have previously observed impaired pulvinar activation to pattern and face stimuli in schizophrenia (Martinez et al., 2019, 2021). Here, we demonstrate similar activation deficits to repetitive flash stimuli (Figures 5C,D). As with the deficit in alpha blocking, the pulvinar activation deficit was equivalent for white and red stimuli, supporting the potential role of magnocellular projections from LGN to the inferior pulvinar nucleus in the mediation of the alpha blocking deficits in schizophrenia during visual stimulation.

### Biomarker utility

Deficits in early visual processing in schizophrenia are extensively characterized using behavioral, electrophysiological and fMRI-based studies (Martinez et al., 2024). Nevertheless, all techniques require specialized equipment and thus cannot be applied outside of dedicated research laboratories. In contrast, flash-VEP may be obtained using off-the-shelf clinical-grade EEG equipment, as in the present study. As expected, the flash-tVEP was increased in schizophrenia especially to red stimuli, reflecting its primary reliance on parvocellular function. In contrast, significant deficits were observed in pattern-tVEP generation within the same participants. Significant deficits were also observed in both the photic driving and alpha blocking responses in EEG. The alpha blocking deficits were mirrored in fMRI by reduced pulvinar activation. The strong correlation between alpha blocking and cognition supports this measure as a target for treatment development.

### Limitations

A limitation of the study is that even through a neutral density filter was used to try to equate luminance across conditions, the matching was only approximate (20% red only vs. 10% cross-colors), so that differences in response could have been driven in part by luminance as well as color differences. Thus, differences could reflect, in part, more general differences in achromatic/luminance vs. chromatic processing, rather than specific magnocellular vs. parvocellular processing. Nevertheless, retinotectal afferents are known to be composed primarily of magnocellular and koniocellular projects from the retina (Grunert et al., 2021; Kim et al., 2021), so differential activation is likely to have limited effect on retinotectal function.

In addition, flicker photometry may provide more accurate differentiation between M, P, and K pathways than flash stimuli as used here (Seraji et al., 2021; Yoshida et al., 2024; Sumner et al., 2002). Such approaches, however, require specialized equipment and thus are inherently less scalable to the clinical setting. Nevertheless, to the extent that flicker photometry has been used to evaluate visual processing in schizophrenia, preferential magnocellular vs. parvocellular dysfunction has been observed (Yoshida et al., 2024).

Last, there was a significant gender imbalance across groups, although the mean value of the ERP and fMRI results did not vary by gender.

## Conclusion

Recent histological studies have shown preferential reductions in NMDAR expression within primary visual cortex in schizophrenia (Schoonover et al., 2024). In this study, using flash-VEP we show a pattern of neurophysiological disturbances that is consistent with dysfunction of NMDAR-mediated neurotransmission within the early visual system as a potential explanation. Deficits include reduced alpha phase-reset to repetitive flash stimuli, reduced alpha blocking, and impaired pulvinar activation to both white and red stimuli. Deficits, moreover, correlate highly with visually sensitive components of neurocognition, including speed of processing and working memory. As opposed to pattern-tVEP, flash-tVEP may be obtained using standard clinical EEG systems, permitting translation of visual biomarkers from research to clinical settings.

## Data Availability

The raw data supporting the conclusions of this article will be made available by the authors, without undue reservation.
