# Supplementary material for: Differential deficits in pattern- versus flash-visual evoked potentials in schizophrenia: relationship to subcortical visual systems, pulvinar nucleus and cognition
Source: Front Neuroimaging. 2026 Jun 18;5:1833352. doi: 10.3389/fnimg.2026.1833352 (PMC13322905; doi:10.3389/fnimg.2026.1833352)
Supplement: Supplementary file 1 [file Supplementary_file_1.docx]

**Supplementary materials**

**Supplementary Figure 1** shows the ongoing intertrial coherence (ITC) within the extracted alpha frequency band (see Figures 1A,C). These data were then used to compute Fast Fourier Transform (FFT) analyses shown in Figures 1B,D. Across groups, the greatest modulation of alpha activity occurred at the 6-Hz stimulation rate, and was larger for white vs. red stimuli.


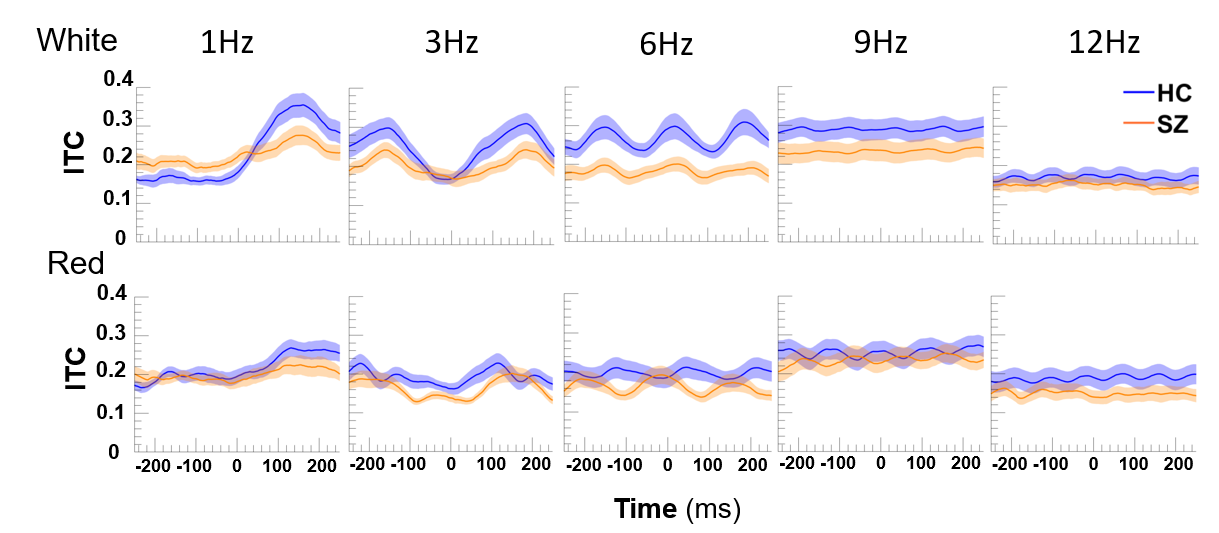


**Supplementary Figure 1**: Intertrial Coherence (ITC) from the extracted alpha frequency at the indicated stimulation rate for healthy control (HC) and schizophrenia (SZ) participants.
